# Efficacy of novel indoor residual spraying methods targeting pyrethroid-resistant *Aedes aegypt*i within experimental houses

**DOI:** 10.1371/journal.pntd.0007203

**Published:** 2019-02-28

**Authors:** Mike W. Dunbar, Fabian Correa-Morales, Felipe Dzul-Manzanilla, Anuar Medina-Barreiro, Wilbert Bibiano-Marín, Evaristo Morales-Ríos, José Vadillo-Sánchez, Beatriz López-Monroy, Scott A. Ritchie, Audrey Lenhart, Pablo Manrique-Saide, Gonzalo M. Vazquez-Prokopec

**Affiliations:** 1 Department of Environmental Sciences, Emory University, Atlanta, Georgia, United States of America; 2 Centro Nacional de Programas Preventivos y Control de Enfermedades (CENAPRECE) Secretaría de Salud México, Ciudad de México, México; 3 Unidad Colaborativa para Bioensayos Entomológicos, Universidad Autónoma de Yucatán, México, México; 4 Universidad Autónoma de Nuevo León, Facultad de Ciencias Biológicas, Nuevo León, México; 5 College of Public Health, Medical & Vet Sciences, James Cook University, Cairns, Australia; 6 Entomology Branch, Division of Parasitic Diseases and Malaria, Centers for Disease Control and Prevention, Atlanta, Georgia, United States of America; University of California, Davis, UNITED STATES

## Abstract

Challenges in maintaining high effectiveness of classic vector control in urban areas has renewed the interest in indoor residual spraying (IRS) as a promising approach for *Aedes*-borne disease prevention. While IRS has many benefits, application time and intrusive indoor applications make its scalability in urban areas difficult. Modifying IRS to account for *Ae*. *aegypti* resting behavior, named targeted IRS (TIRS, spraying walls below 1.5 m and under furniture) can reduce application time; however, an untested assumption is that modifications to IRS will not negatively impact entomological efficacy. We conducted a comparative experimental study evaluating the residual efficacy of classically-applied IRS (as developed for malaria control) compared to two TIRS application methods using a carbamate insecticide against a pyrethroid-resistant, field-derived *Ae*. *aegypti* strain. We performed our study within a novel experimental house setting (n = 9 houses) located in Merida (Mexico), with similar layouts and standardized contents. Classic IRS application (insecticide applied to full walls and under furniture) was compared to: a) TIRS: insecticide applied to walls below 1.5 m and under furniture, and b) Resting Site TIRS (RS-TIRS): insecticide applied only under furniture. Mosquito mortality was measured eight times post-application (out to six months post-application) by releasing 100 *Ae*. *aegypti* females /house and collecting live and dead individuals after 24 hrs exposure. Compared to Classic IRS, TIRS and RS-TIRS took less time to apply (31% and 82% reduction, respectively) and used less insecticide (38% and 85% reduction, respectively). Mortality of pyrethroid-resistant *Ae*. *aegypti* did not significantly differ among the three IRS application methods up to two months post application, and did not significantly differ between Classic IRS and TIRS up to four months post application. These data illustrate that optimizing IRS to more efficiently target *Ae*. *aegypti* can both reduce application time and insecticide volume with no apparent reduction in entomological efficacy.

## Author summary

Vector control is the primary strategy for managing *Aedes aegypti* and reducing transmission of *Aedes*-borne diseases; however, the indoor resting behavior of *Ae*. *aegypti* and the evolution of insecticide resistance reduces the effectiveness of many vector control tactics. Indoor residual spraying (IRS) is effective against *Ae*. *aegypti*, but lengthy application time makes IRS difficult to scale within urban environments. We compared the application and entomological efficacy of Classic IRS against two novel *Aedes*-targeting IRS application methods (Targeted IRS [TIRS]- insecticide applied to walls below 1.5 m and under furniture and Resting Site TIRS [RS-TIRS]- insecticide applied only under furniture) within experimental houses using a carbamate insecticide. Both TIRS and RS-TIRS took less time to apply and used less insecticide compared to Classic IRS. Mortality of pyrethroid-resistant *Ae*. *aegypti* did not differ among treatments out to two months post-application, and there was no difference in mortality between Classic IRS and TIRS out to four months post-application. These data provide evidence that IRS application methods can be improved to take less time and insecticide yet not lose entomological efficacy, making TIRS more scalable within urban environments. However, larger field studies with epidemiologic endpoints are needed to further assess the efficacy of these modified TIRS techniques.

## Introduction

Vector control is the principal approach for managing *Aedes aegypti* and reducing transmission of *Aedes*-borne diseases (ABD; *e*.*g*., dengue, chikungunya, Zika). Implementation of vector control targeting ABDs has primarily been in response to reports of virus transmission, using methods such as truck-mounted ultra-low volume spraying (ULV)/thermal fogging, source reduction and larviciding [1, 2]. Recent assessments of the public health value of these reactive interventions, triggered by the need to contain Zika transmission and prevent the devastating congenital malformations attributed to infection of pregnant woman, has highlighted the dearth of data supporting the role of vector control tactics in preventing ABDs [3–5]. Multiple factors challenge the efficacy and coverage of existing vector control tactics, including rapid urbanization leading to widespread *Ae*. *aegypti* distribution [6], the occurrence of cryptic larval habitats [7, 8], the rapid rise of insecticide resistance [9] and the multiplicity of virus transmission locations generated by fine-scale human mobility patterns [10, 11]. Given these challenges, management of *Ae*. *aegypti* requires highly effective, innovative approaches that can be implemented across epidemiological settings and within integrated vector management strategies [4].

Adult *Ae*. *aegypti* in urban settings typically rest indoors, where they feed frequently and almost exclusively on human blood [12–14]. This endophilic and anthropophilic behavior partially explains why outdoor space spraying (*e*.*g*., truck-mounted ultra-low volume spraying) has very limited efficacy against *Ae*. *aegypti* and ABD transmission [15]. Vector control methods that deliver insecticides indoors are more promising because they can exert a direct impact on resting adult mosquitoes [5]. The principal methods of applying insecticides indoors are indoor space spraying (ISS; application of insecticides with a droplet size of < 50 μm that kill adult vectors upon contact [5]) and indoor residual spraying (IRS; the application of aqueous formulations of insecticides with longer term residual efficacy on the walls and ceilings of houses that kill the adult vectors landing on these surfaces [16]). In terms of application and performance, ISS and IRS are very different. Indoor space spraying can be deployed rapidly, particularly during epidemics, because it can be applied quickly (< 10 min), but ISS can require up to three application cycles to achieve maximum efficacy and has a short-lived insecticidal effect, as it only targets flying mosquitoes making contact with the transient insecticidal cloud. Indoor residual spraying can provide longer-term protection after a single application; however, application time can be lengthy if all furniture and belongings need to be removed from the spray area. Despite field evidence pointing to significant epidemiological impacts of IRS in preventing dengue [5, 10, 17], and recent modeling work forecasting significant long-term reductions in disease burden after its implementation [18], the perceived labor-intensive nature of IRS (in comparison to ISS) and issues of community acceptance [19] have hindered its adoption for urban vector control targeting *Ae*. *aegypti*.

To overcome the time-consuming aspects of IRS and account for *Ae*. *aegypti*-specific behaviors, several modifications to the ‘classic’ IRS strategy intended to control vectors of malaria or Chagas disease (*i*.*e*., full house spraying, movement of furniture and treatment of all walls and ceiling) have been proposed. In Cairns, Australia, IRS is performed targeting *Ae*. *aegypti* resting sites, and insecticide is applied to exposed low walls (below 1.5 m), under furniture, inside closets and on any dark and moist surface where *Ae*. *aegypti* may be found resting [10]. This modified IRS was implemented in Cairns after the detection of local dengue transmission and dramatically reduced IRS application time and resulted in the successful containment of multiple outbreaks [10, 17, 20].

One of the untested assumptions of the modifications introduced to the classically-applied IRS is that there is no negative impact on entomological efficacy. Using a novel experimental house setting, we conducted a comparative study to evaluate the residual efficacy of classically-applied IRS against two novel IRS application methods using a non-pyrethroid insecticide against a locally-derived, pyrethroid-resistant strain of *Ae*. *aegypti*. For each IRS application method, the application time and volume of insecticide used were measured. Entomological impact over time was compared among the IRS application methods. We hypothesized that the two novel IRS application methods would provide similar levels of entomological efficacy as classically-applied IRS, but would be applied faster and use less insecticide. Furthermore, we hypothesized that the efficacy of a non-pyrethroid insecticide, specifically a carbamate insecticide (bendiocarb), would be similar between the two novel IRS application methods and classically-applied IRS.

## Methods

### Experimental design

Within a replicated system of nine experimental houses, we tested the residual efficacy of three IRS application methods on free flying, field-derived *Ae*. *aegypti*. The experimental houses were located in Caucel, a neighborhood at the periphery of the subtropical city of Mérida, México, and were rented long-term by the Universidad Autónoma de Yucatán (UADY) after explaining the purpose and extent of the study to the owners. Mérida is the capital of the state of Yucatán, has a population of roughly one million and experiences a rainy season from May through October. Dengue is endemic and transmission occurs throughout the year, although peak transmission occurs between July and November and corresponds with the rainy season [18, 21, 22]. Average dengue sero-prevalence rate in the population is 73.6% [23]. Since 2016, Chikungunya and Zika viruses also circulate within Merida, impacting the public health system and vector control operations [22]. Local management tactics for *Ae*. *aegypti* include ISS with either pyrethroids (*e*.*g*., deltamethrin) or organophosphates (*e*.*g*., malathion) and ULV with organophosphate insecticides (*e*.*g*., chlorpyrifos and malathion) [24]. Resistance to pyrethroids (both type I and type II) occurs in local *Ae*. *aegypti* populations, however these populations are still presently susceptible to carbamates [24–26].

**Fig 1:**
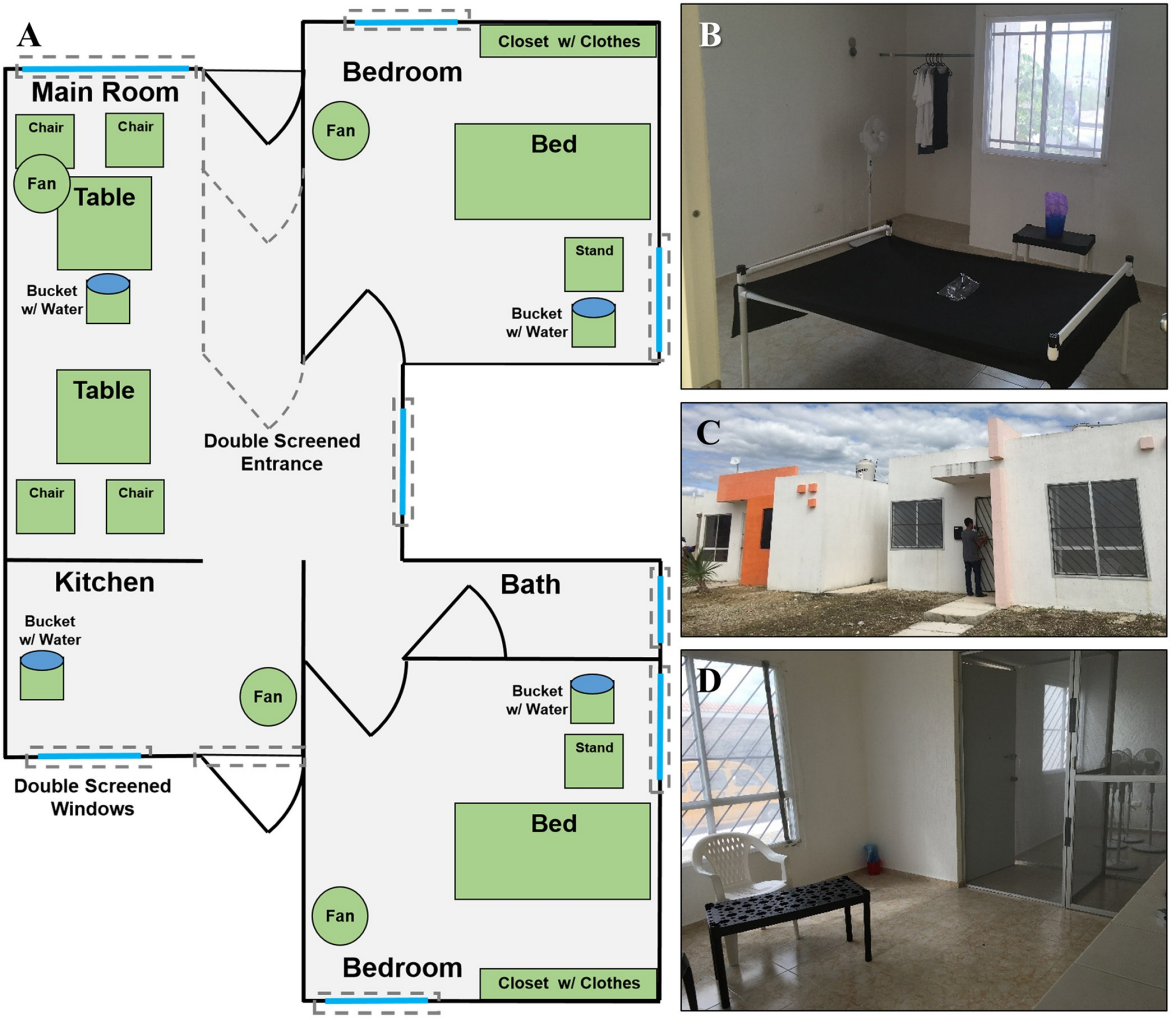
Layout of experimental houses. (A) General layout, (B) setup of bedrooms, (C) exterior entrance, and (D) living room and double-screened entrance of experimental houses.

Distance between experimental houses ranged from 0.3 to 2 km. The houses were similar in floor plan and design; all were concrete, single-story and had one or two living rooms, two bedrooms, one bathroom and one kitchen (Fig 1). Houses were on average 57.8 ± 2.8 m^2^ (mean ± SEM) and uniformly had walls 2.5 m in height. Construction characteristics were that of subsidized middle to low-income housing in Mérida, typical of areas with high ABD transmission [22].

To prevent any mosquitoes used in the experiments from escaping from the houses, all windows and doors were screened on both the outside and inside of each house before the study began. Additionally, a double screened-door vestibule was built into the main entrance of each house to allow personnel to enter and exit while preventing mosquitoes from escaping (Fig 1). Sinks, drains and toilets were also sealed with window screening. Existing furniture within houses was removed, and where furniture could not be removed (*e*.*g*., built-in kitchen or closet cabinets) it was sealed with window screening. Houses were then refurnished with standardized furniture and clothing that represented typical elements found within houses (Fig 1). Furniture within in the living room (or split between two living rooms) included two black plastic tables and four plastic chairs. Within each bedroom was a bed made out of PVC tubing and black cloth, a black plastic night stand and six articles of clothing (3 black and 3 white) hung within the closet. Additionally, four plastic buckets (1 L) were half filled with water and a dark cloth and placed throughout each house to provide moisture into the environment and reduce mosquito mortality due to desiccation. Ant baits (Antex Gel, Allister de México) were placed next to each door or any other location where ants were observed to enter the experimental houses. The house layout was carefully designed to mirror elements and surface materials found in regular homes, but making sure that they were standardized in a way that allowed replication and comparability between replicates.

### Insecticide application

Insecticide was applied within experimental houses on 3 July 2017. A manual compression sprayer (Hudson 93793 X-Pert) fitted with flat nozzles and a flow control valve (model CFV.R11/16SYV.ST, CFValue, Gate LLC) was used to spray houses at a flow rate of 550 mL / min. Bendiocarb (Ficam 80% WP, Bayer CropScience; 125 g sachet / 7.5 L water), a carbamate insecticide, was applied at a dosage of 0.375 g active ingredient / m^2^ as recommended by the WHO [16]. Bendiocarb was used because of the known susceptibility of local *Ae*. *aegypti* populations that were resistant to synthetic pyrethroids [24]. Additionally, a previous RCT in Mérida found high community acceptance of bendiocarb, with no reported adverse reactions, when it had been applied within homes [24]. The same individual applied insecticide for each of the nine experimental houses.

Houses were randomly assigned to one of three different IRS application methods: 1) Classic IRS- insecticide applied to walls and under furniture (n = 3 houses), 2) Targeted IRS (TIRS)- insecticide applied to walls below 1.5 m and under furniture (n = 3 houses) or 3) Resting Site TIRS (RS-TIRS)- insecticide only applied under furniture (n = 3 houses). Furniture was not removed from experimental houses during the insecticide application and insecticide was not applied to clothing or the plastic buckets with water. Duration of application was measured for each house, starting when the applicator entered the house and ending when the applicator exited. To estimate the volume of insecticide applied within each house, the insecticide within the sprayer was measured using a graduated cylinder before and after each application.

### Mosquito strain

To test the residual efficacy of each IRS application method, a total of 100 *Ae*. *aegypti* females were released within each experimental house. The strain used (San Lorenzo strain) was locally derived, had a high level of resistance to pyrethroids and full susceptibility to carbamates [24, 26]. The San Lorenzo strain was reared and maintained at the insectaries of the Unidad Colaborativa para Bioensayos Entomológicos, UADY, Mérida, México. Mosquitoes released into houses were three to seven days old from the F4 generation, before release had only been provided sugar solution and were non-bloodfed.

Post-insecticide application, mosquitoes were released into the experimental houses eight times over a six month period; 1) +1 day, 2) +14 days, 3) +1 month, 4) +2 months, 5) +3 months, 6) +4 months, 7) +5 months and 8) +6 months. To facilitate mosquito recovery, all experimental houses were vacuumed and swept clean of any debris on the floor one day prior to mosquito release. After 24 hrs exposure, a team of four field technicians entered each house and searched for live mosquitoes using a Prokopack aspirator [27] and searched by hand for dead mosquitoes. Searching for *Ae*. *aegypti* ceased when either 100 mosquitoes were collected or > 20 minutes elapsed after the last mosquito was collected (circa 30–40 min / house). Natural mortality within experimental houses was measured by placing three unsprayed control cups (250 mL) within each house, with each cup containing 10 San Lorenzo strain females. Control cups were placed within experimental houses simultaneously during the main release of mosquitoes during the +4, +5 and +6 months post-application evaluations. After searching for released *Ae*. *aegypti* ceased, the number of live and dead *Ae*. *aegypti* within control cups were counted.

### Statistical analyses

For each sampling period, mortality was calculated per house by dividing the number of dead individuals by the number of individuals released. Missing individuals were assumed to be dead. Mortality was compared between IRS application methods using mixed-model analysis of variance (ANOVA) in R 3.2 statistical software (https://www.r-project.org/). Sampling date, IRS application method, and their interaction were classified as fixed effects and experimental house was classified as a random effect. When significant differences were detected, pairwise comparisons were made using LSMEAN package and alpha levels were adjusted for multiple comparisons using the Tukey correction. Additionally, regression analysis was used to assess the relationship between application time and volume of insecticide applied among the three IRS application methods.

### Ethics statement

This was an experimental study, and because mosquitoes were released into uninhabited houses rented on long-term contracts, we did not require an Institutional Review Board.

## Results

### Insecticide application

Compared to Classic IRS, TIRS reduced application time on average by 5.8 min / house (31.3% reduction), whereas RS-TIRS reduced application time on average by 15.2 min / house (82.0% reduction) (Table 1). Similarly, compared to Classic IRS, TIRS used on average 2.02 L / house less insecticide (37.9% reduction), while RS-TIRS saved on average 4.53 L / house (84.8% reduction) (Table 1). Compared to TIRS, RS-TIRS reduced both application time by 9.40 min / house (73.8% reduction) and insecticide volume by 2.50 L / house (75.5% reduction) (Table 1). Reductions in both application time and insecticide volume were significantly linear (*F* = 140.1; df = 1, 7; *P* < 0.0001), indicating consistent insecticide application among IRS application methods.

**Table 1:** Application time and volume of insecticide applied within experimental houses for the three IRS application modes.

Application Method	Total Area^a^ Range (Mean % Treated)	Application Time (min)	Volume Applied (L)
Classic IRS	137–141 m^2^ (100 ± 0%)^b^	18.6 ± 3.1^b^	5.34 ± 0.58^b^
Targeted IRS	137–173 m^2^ (65.8 ± 0.6%)	12.7 ± 1.1	3.32 ± 0.18
Resting Site Targeted IRS	160–171 m^2^ (5.9 ± 0.1%)	3.3 ± 0.4	0.81 ± 0.11

^a^ Total area = sum area of walls plus the area of the furniture within the experimental house.

^b^ Mean ± Standard error of the mean

### Mosquito recovery and mortality

A total of 7,200 *Ae*. *aegypti* females were released within the experimental houses throughout the trial. Mosquito recovery averaged 96.9 ± 0.82% (Mean ± SEM; n = 72 releases). Based on pilot data, we attribute high recovery to pre-cleaning the floors of experimental houses the day before mosquitoes were released and to effective management of ants using baits.

Mortality within control cups average 4.4 ± 1.3%, 1.5 ± 0.7% and 5.0 ± 1.7% (Mean ± SEM) for evaluations from +4, +5 and +6 months post-application, respectively, indicating high *Ae*. *aegypti* survival within the experimental house environments.

**Fig 2:**
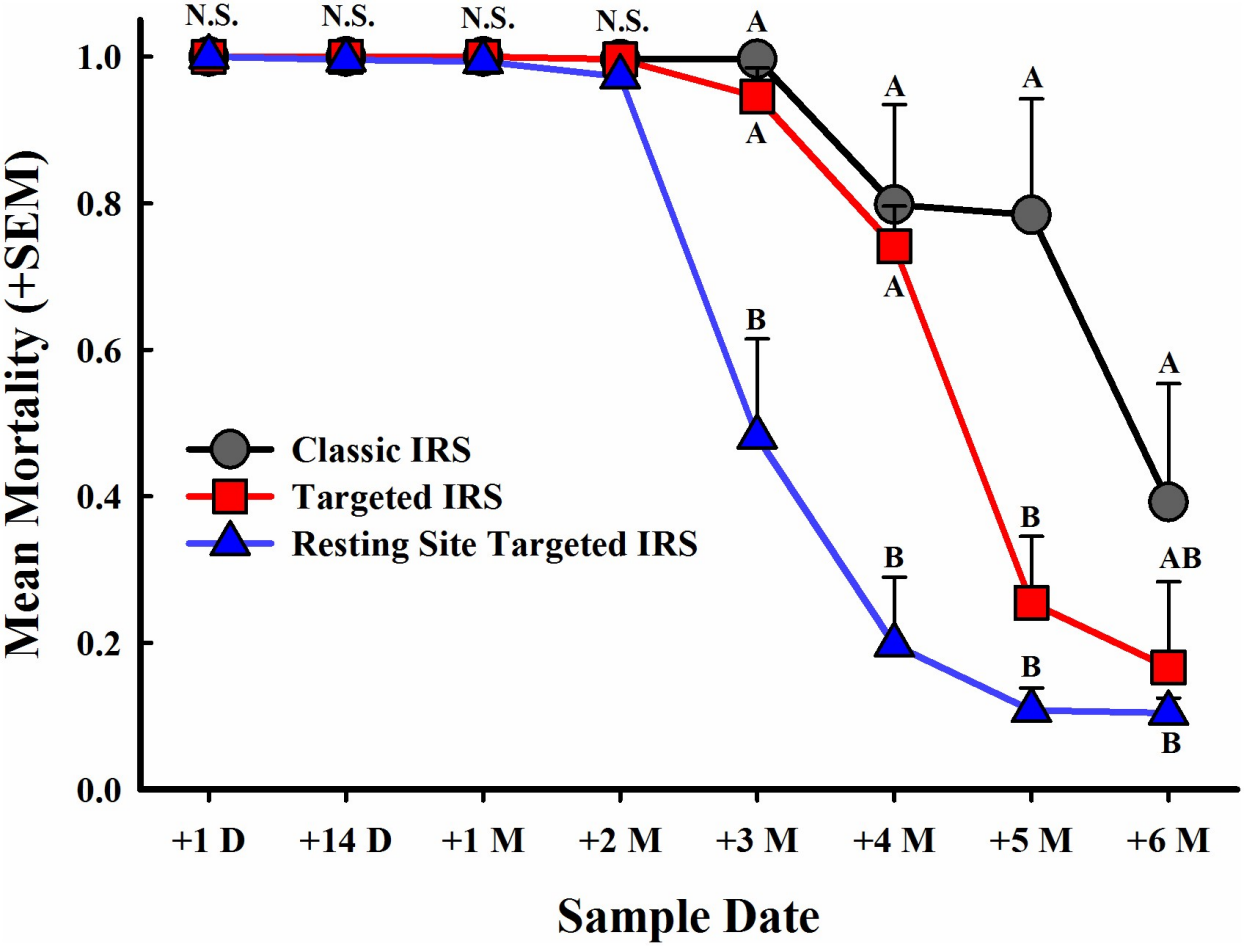
Mortality of pyrethroid-resistant *Ae*. *aegypti* by IRS application method using bendiocarb over time. Symbols denote sample means and error bars are the standard error of the mean. Letters denote significant differences among IRS application methods within sample date.

There was a significant interaction between IRS application method and sampling time post application (*F* = 6.3; df = 14, 42; *P* < 0.0001) (Fig 2). Almost complete mortality of all released mosquitoes was observed up to two months post-application (ranging from 97.3 to 100%); there were no significant differences in mortality among the three IRS treatments within the first 4 sampling periods. At three months post-application, mortality of *Ae*. *aegypti* dropped significantly in houses treated with RS-TIRS (from 97.3% at +2 months to 48.1% at +3 months) compared to Classic IRS and TIRS houses, where mortality remained high (99.7% and 94.5%, respectively). At four months post-application, mortality of *Ae*. *aegypti* from Classic IRS and TIRS treated houses dropped to 79.8% and 74.2%, respectively, but were both significantly greater compared to mortality of *Ae*. *aegypti* from RS-TIRS houses, which dropped to 19.7%. Mortality in experimental houses with Classic IRS remained high five months post-application (78.4%) and was significantly greater compared to both TIRS (25.5%) and RS-TIRS (10.8%), which did not differ from each other. Efficacy of all three treatments was greatly reduced six months post-application (one month beyond the expected residual duration of bendiocarb). Mortality in Classic IRS treated houses was reduced to 39.2%, yet was significantly greater compared to RS-TIRS (10.4%), although neither treatment differed significantly from TIRS (16.6%) (Fig 2).

## Discussion

We compared the residual efficacy of Classic IRS against two novel IRS application methods, TIRS and RS-TIRS, in experimental houses, and hypothesized that the two novel IRS application methods would be as efficacious as Classic IRS. Furthermore, we hypothesized that the efficacy of a non-pyrethroid insecticide, bendiocarb, would be similar among the two novel IRS application methods and Classic IRS. Although both TIRS and RS-TIRS took less time to apply and used less insecticide compared to Classis IRS (Table 1), these data support our hypotheses, as pyrethroid-resistant *Ae*. *aegypti* mortality did not differ among the three IRS application methods up to two months post-application and did not differ between Classic IRS and TIRS up to four months post-application (Fig 2).

Using bioassays within experimental houses that closely simulate typical living conditions, this study provides important information that can help improve the mode of IRS application and cost-effectiveness within the urban context of ABD transmission. Improvements in IRS efficiency and application are key for increasing scalability and adoption of this management tactic [28]. Recent and rapid scaling-up of IRS for malaria control illustrate the potential public health benefits of this approach [29], but also point to the difficulties of reaching and sustaining high coverage levels due to IRS’s labor-intensive nature [30]. If IRS were to be widely adopted for urban *Ae*. *aegypti* management, lessons from IRS scale-up for malaria vector control should be taken into consideration to better frame the operational conditions and approaches for intervention delivery.

Field observational studies from Central and South America have found that *Ae*. *aegypti* primarily rest indoors and below 1.5 m, particularly on or near dark places such as behind or under furniture, under beds, on clothing and on lower parts of walls [13, 27, 31]. This low-resting behavior has also been observed in experimental hut studies using an *Ae*. *aegypti* strain from Thailand [32]. Modifying IRS to account for key *Ae*. *aegypti* resting behaviors resulted in important reductions in application time and insecticide volume (Table 1) without sacrificing entomological efficacy for two to four months post application (Fig 2). The fact that we detected high mortality with no statistical difference between Classic IRS and TIRS methods show that *Ae*. *aegypti* are not avoiding treated locations by shifting resting behaviors above 1.5 m. Additionally, RS-TIRS was applied only to common resting sites (beds, chairs and other furniture) and resulted in to up to 2 months of full protection, providing further evidence of the remarkable preference of *Ae*. *aegypti* for specific resting locations.

Duration of protection differed between TIRS and RS-TIRS applications. Although RS-TIRS could be completed on average in 3.3 min / house (Table 1), the protection provided (using > 80% mortality as a threshold) by this approach lasted two months, or half the duration of Classic IRS or TIRS (Fig 2). One of the challenges of RS-TIRS when applied in real households (which would likely be more cluttered and full of personal items than our experimental houses) is that it may entail the treatment of personal belongings that are preferentially used by *Ae*. *aegypti* as resting sites (*e*.*g*., suitcases, clothes, etc.). Applying insecticide to personal belongings could potentially lead to community disapproval of the methodology, as well as potentially result in unanticipated exposure to insecticides [19]. As such, while there are significant reductions in application time and insecticide volume, performing RS-TIRS may be more challenging than performing TIRS. Given that TIRS provides longer-term protection (up to 4 months) compared to RS-TIRS, we see the former as a methodology highly suitable for implementation within the context of urban *Ae*. *aegypti* management.

A randomized controlled trial evaluating the entomological impact of Classic IRS using bendiocarb against pyrethroid-resistant populations of *Ae*. *aegypti* in Mérida, México, demonstrated a 65–75% reduction in adult *Ae*. *aegypti* abundance in treatment clusters, compared to controls, up to three months post-application [24]. Furthermore, the application time of Classic IRS from this trial averaged approximately 30 min / house [24]. Our experimental study demonstrated that an application of TIRS required roughly 12 min to complete but resulted in a 4-month protection of treated houses. The residual effects observed were driven by the insecticide used (bendiocarb residuality is expected to last between 3 and 5 months), and its interaction with treated substrates (in our case, painted walls, cloth, wood and plastic). Given the recent development of new residual insecticide formulations for malaria, which extend residual duration out to 6–8 months and are effective against pyrethroid-resistant mosquitoes [33, 34], there is potential for extending residual power of TIRS beyond the 4-month mark.

Despite the higher cost of novel insecticide formulations, applying novel insecticides via TIRS would not only reduce application time but also potentially increase cost-effectiveness. Furthermore, extending residual duration can provide a longer window of protection and shift IRS application from reactive (in response to reported clinical cases, as in [10]) to pro-active (performed prior to the transmission season [18]). A recent analysis of historical dengue, chikungunya and Zika cases geocoded to the household level found a significant level of spatial overlap of the three pathogens within specific geographic units that accumulated more than half of all cases [22]. The pro-active (pre-season) deployment of high-quality interventions such as TIRS within hot-spot areas could offer additional protection to areas that consistently report high rates of ABD transmission [22, 35]. An insecticide with residual duration that lasts more than 5 months could protect a household for an entire transmission season (which lasts 5 to 6 months) using a single TIRS application. Additionally, using insecticides pro-actively should be coupled with insecticide-resistance monitoring and insecticides used for TIRS changed when resistance is first detected. Previous studies have demonstrated that fitness costs associated with pyrethroid resistance in *Aedes aegypti* do exist and that susceptibility can be regained in the absence of selection [36]. While the efficacy of such pro-active TIRS implementation in preventing ABD will require further evaluations with proper epidemiologic endpoints [37], the findings presented here provide clear evidence for how IRS applications could be optimized for urban *Aedes* management. However, larger field studies with epidemiologic endpoints are needed to further assess the efficacy of these modified TIRS techniques.
